# Frequency and Significance of Pathologic Pulmonary Findings in Postmortem Examinations—A Single Center Experience before COVID-19

**DOI:** 10.3390/diagnostics11050894

**Published:** 2021-05-18

**Authors:** Sabina Berezowska, Andreas Schmid, Tereza Losmanová, Mafalda Trippel, Annika Blank, Yara Banz, Stephan M. Jakob, Rupert Langer

**Affiliations:** 1 Institute of Pathology, University of Bern, Murtenstrasse 31, 3008 Bern, Switzerland; sabina.berezowska@chuv.ch (S.B.); andreas.schmid3@gmx.ch (A.S.); losmanova.t@centrum.cz (T.L.); mafalda.trippel@pathology.unibe.ch (M.T.); Annika.blank@triemli.zuerich.ch (A.B.); yara.banz@pathology.unibe.ch (Y.B.); 2 Department of Laboratory Medicine and Pathology, Institute of Pathology, Lausanne University Hospital, University of Lausanne, Rue du Bugnon 25, 1011 Lausanne, Switzerland; 3 Department of Intensive Care Medicine, Inselspital, Bern University Hospital, Freiburgstrasse 18, 3010 Bern, Switzerland; stephan.jakob@insel.ch; 4 Institute of Pathology and Molecular Pathology, Kepler University Hospital, Johannes Kepler University Linz, Krankenhausstr 9, 4021 Linz, Austria

**Keywords:** pulmonary pathology, postmortem diagnostics, autopsy

## Abstract

Coronavirus disease 2019 (COVID-19) has shown the importance of postmortem investigation of deceased patients. For a correct interpretation of the pulmonary findings in this new era, it is, however, crucial to be familiar with pathologic pulmonary conditions observed in postmortem investigations in general. Adequate postmortem histopathological evaluation of the lungs may be affected by suboptimal gross work up, autolysis or poor fixation. Using a standardized preparation approach which consisted in instillation of 4% buffered formaldehyde through the large bronchi for proper fixation and preparing large frontal tissue sections of 1–2 cm thickness after at least 24 h fixation, we comprehensively analyzed postmortem pulmonary findings from consecutive adult autopsies of a two-year period before the occurrence of COVID-19 (2016–2017). In total, significant pathological findings were observed in 97/189 patients (51%), with 28 patients showing more than one pathologic condition. Acute pneumonia was diagnosed 33/128 times (26%), embolism 24 times (19%), primary pulmonary neoplasms 18 times (14%), organizing pneumonia and other fibrosing conditions 14 times (11%), pulmonary metastases 13 times (10%), diffuse alveolar damage 12 times (9%), severe emphysema 9 times (7%) and other pathologies, e.g., amyloidosis 5/128 times (4%). Pulmonary/cardiopulmonary disease was the cause of death in 60 patients (32%). Clinical and pathological diagnoses regarding lung findings correlated completely in 75 patients (40%). Autopsy led to confirmation of a clinically suspected pulmonary diagnosis in 57 patients (39%) and clarification of an unclear clinical lung finding in 16 patients (8%). Major discrepant findings regarding the lungs (N = 31; 16%) comprised cases with clinical suspicions that could not be confirmed or new findings not diagnosed *intra vitam*. A significant proportion of acute pneumonias (N = 8; 24% of all cases with this diagnosis; *p* = 0.011) was not diagnosed clinically. We confirmed the frequent occurrence of pulmonary pathologies in autopsies, including inflammatory and neoplastic lesions as the most frequent pathological findings. Acute pneumonia was an important cause for discrepancy between clinical and postmortem diagnostics

## 1. Introduction

The recent advent of Coronavirus disease 2019 (COVID-19) has turned the focus on pulmonary findings of deceased patients. Postmortem investigations of the lungs of COVID-19 patients have contributed significantly to the understanding of this disease [1,2,3,4]. Apart from this recent development, pathological pulmonary findings in adults are frequently encountered at postmortem examinations, either as the leading cause of death [5,6,7,8,9,10,11,12,13] or as a secondary significant finding, however unrelated to the cause of death. They comprise inflammatory or immunologic conditions, primary or secondary tumors, fibrotic changes or degenerative, often emphysematous conditions. Inflammatory and fibrotic changes may be challenging to categorize clinically, and autopsies may confirm or contradict a proposed clinical diagnosis. Moreover, discrepancy between clinical and postmortem pathologic findings is well-known [6,7,8,9,10,11,12,13].

Despite a large amount of literature on pulmonary findings in postmortem diagnostics, including a vast number of case reports (>5100 hits for PubMed search: “case report” and “pulmonary pathology” and “autopsy” [14]) and epidemiologically oriented studies examining the cause of death [5], data focusing particularly on pulmonary findings obtained from larger autopsy series—apart from recent COVID-19 publications—are surprisingly scarce. Knowledge about autoptic pulmonary pathology in general, however, is a crucial prerequisite for a reliable diagnostic process, in particular when faced with the task of describing a novel disease as the world has experienced it dramatically in 2020.

Postmortem analysis of the lungs may be affected by several pre-analytical factors including suboptimal gross work up, autolysis or poor fixation, which particularly impedes proper histopathological evaluation. Our postmortem lung work-up was revised and standardized within the context of restructuring the autopsy department of the Institute of Pathology of the University of Bern to a “Postmortem Diagnostic (PMD) Unit” as described in detail elsewhere [15], consisting in instillation of formaldehyde through the large bronchi and preparing large frontal tissue sections after proper fixation.

In order to gain a more comprehensive overview of postmortem pulmonary pathology findings, we prospectively collected all data on lung pathology findings obtained from consecutive adult autopsies during a period of two years using this standardized approach and compared them with clinical diagnoses.

## 2. Materials and Methods

### 2.1. Postmortem Diagnostic Procedure

**Figure 1:**
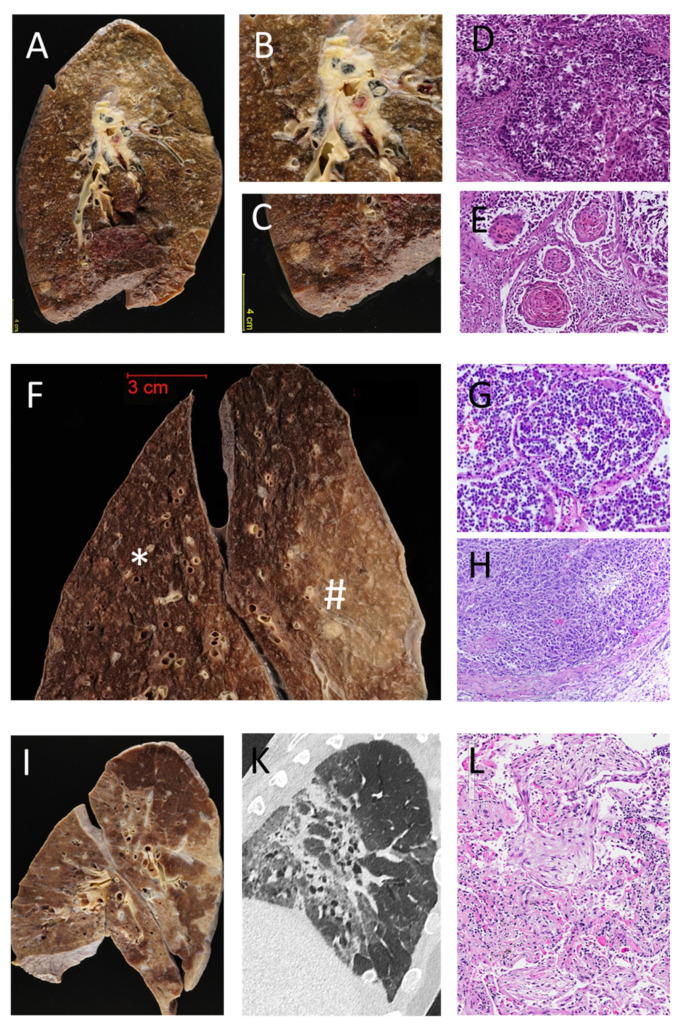
Illustrative examples of postmortem findings in the lungs: (**A**–**E**) Case 1: Clinical Diagnosis: pulmonary bleeding, most probably due to a central lung cancer. (**A**) Large transversal section shows a large bronchial carcinoma, centrally located with infiltration and with arrosion of large vessels and bronchi (**A**,**B**). Additionally, in the lower lobe, a second tumor was identified (**A**,**C**). Histologically, the central tumor was a poorly differentiated squamous cell carcinoma (**D**). The tumor in the lower lobe (**E**) was a well differentiated squamous cell carcinoma, due to the different morphology considered to be a second, independent malignancy. (**F**–**H**) Case 2: Clinical Diagnosis: diffuse metastasizing melanoma. F: multiple, partially inhomogeneous (*), partially well circumscribed (#) nodules and indurations in the lungs. Histological coexistence of acute bronchopneumonia (**G**, corresponding to *) and multiple intravascular infiltrations of melanoma (**H**, corresponding to #). No parenchymatous metastases were detected. (**I**–**L**) Case 3: Clinical Diagnosis: unclear pulmonary fibrosis. (**I**): Patchy consolidations in all lobes. (**K**) Corresponding CT scan. Histology showed organizing pneumonia (**L**). All histological stains are Hematoxylin/ and Eosin; original magnification 20×.

Both lungs were removed after evisceration of the heart and evaluation of the large pulmonary arteries for central pulmonary emboli. Four percent buffered formaldehyde was instilled through the large bronchi using a large 60 mL syringe [16]. After fixation for at least 24 h, large frontal tissue sections of 1–2 cm thickness were prepared using a support device tailor-made for the purpose of lung sectioning. This approach allows complete expansion of the parenchyma and prevents artifacts due to dystelectases or poor fixation. In contrast to the original description [16], we decided to perform fixation for a longer period than one hour. After sectioning, the parenchyma was carefully evaluated. Standardized histology samples were taken from every lobe and from macroscopically abnormal areas, if identified. Histological stains comprised hematoxylin-eosin for all samples and additional Periodic acid-Schiff (PAS) reaction, Elastica van Gieson (EvG) and Iron stain on at least one tissue block. Grocott’s methenamine silver (GMS) staining was only performed in cases with suspicion of fungal infection if PAS staining was negative (Figure 1 shows representative macroscopic and histologic images of selected cases).

### 2.2. Patients

During the period of two years after the introduction of the new technique in January 2016, data from all adult autopsy cases (patients 18 years and older; N = 189; 2016–2017) were prospectively collected. The findings were retrospectively correlated with clinical questions regarding pulmonary pathology and discrepancies between pathology and clinical findings were recorded. The study was performed in accordance with the Swiss Research Act as confirmed by the Ethics commission of the Canton Bern (2017-01189).

### 2.3. Data Recording

Demographic analysis, gender and age of the deceased patients were recorded, as well as the requesting hospital and department (Intensive Care Unit of the University Hospital, other departments of the University Hospital, peripheral hospitals and privately commissioned autopsies). In line with literature [17,18], the cause of death was categorized into sepsis/peritonitis, cardiopulmonary failure, cerebrovascular lesion, pulmonary embolism, pneumonia, myocardial infarction, gastrointestinal hemorrhage, hepatic failure, intestinal ischemia, malignancy, aortic rupture/cardiac tamponade and other. Pulmonary findings were classified into normal, congestion, mild emphysema, diffuse alveolar damage (DAD), acute pneumonia, organizing pneumonia, primary pulmonary neoplasm, pulmonary metastases, central pulmonary embolism, paracentral and peripheral embolism, fibrosis other than organizing pneumonia, severe emphysema (defined as Grade 4 and more according to Nagai et al. [19]) or other pathology. This categorization was used both for the predominant lung findings and for additional pulmonary pathologies if present. Heart weight and lung weight prior to fixation were recorded. Clinical questions listed in the request documents regarding pulmonary findings were categorized as follows: no question, primary tumor, secondary tumor (metastasis), inflammation, degeneration including emphysema, fibrosis. The correlation with clinical questions regarding the lungs was defined as consistent, specific clinical question clarified by postmortem examination (e.g., clinically suspected pulmonary embolism), unspecific or vague clinical question answered or clarified by postmortem examination (e.g., unclear opacity), clinical suspicion of specific finding but discrepant finding in postmortem examination, finding in postmortem examination not suspected clinically. 

### 2.4. Statistics 

The IBM SPSS software V26 (SPSS Inc., Chicago, IL, USA) was used for statistical analysis. Descriptive analyses were performed using simple cross tables. Comparison between groups was performed using Chi-square tests and Fisher’s exact tests and Median tests. *p*-values of <0.05 were considered statistically significant.

## 3. Results

### 3.1. Patient Characteristics and Causes of Death

Postmortem procedures were conducted on 189 consecutive adult patients. A total of 63 (33%) were women and 126 (66%) men. The median age was 69 years (range: 26–104). All postmortem examinations consisted of diagnostic autopsies, excluding minimally invasive approaches where the described method of lung preparation was technically not feasible. Thirty-two (17%) autopsies were from the intensive care unit and 34 (18%) from other departments of the University Hospital Bern (Inselspital). Most autopsies (119; 63%) were solicited by community hospitals, and four (2%) autopsies were commissioned by others (general practitioners, relatives). The most frequent cause of death was cardio-pulmonary failure (N = 54; 29%), including 18 cases (10%) with a predominant respiratory component (DAD, N = 7; severe emphysema, N = 7; fibrosis, N = 4), followed by malignant neoplasm (N = 27; 14%), including 12 cases with primary lung cancer (3%), myocardial infarction (N = 21; 11%), pneumonia (N = 20; 11%), sepsis or peritonitis (N = 12; 6%), aortic rupture (N = 11; 6%), gastrointestinal hemorrhage (N = 11; 6%) and pulmonary embolism (N = 10; 5%). Less frequent causes of death were cerebral and cerebrovascular conditions (N = 7; 4%), hepatic failure (N = 6; 3%), intestinal ischemia (N = 2; 1%) and others (N = 8; 4%). In summary, pulmonary pathologies predominantly contributed to 60/189 deaths (32%) (Table 1).

Lung specific clinical questions had been asked in the autopsy request forms in 104 cases (55%), in particular with regard to neoplasms (N = 32; 17%), pneumonia (N = 19; 10%), emphysema (N = 24; 13%) and idiopathic pulmonary fibrosis (N = 1). In 28 cases (15%), the clinical findings in the lungs were stated as unclear.

### 3.2. Pathological Pulmonary Findings

**Figure 2:**
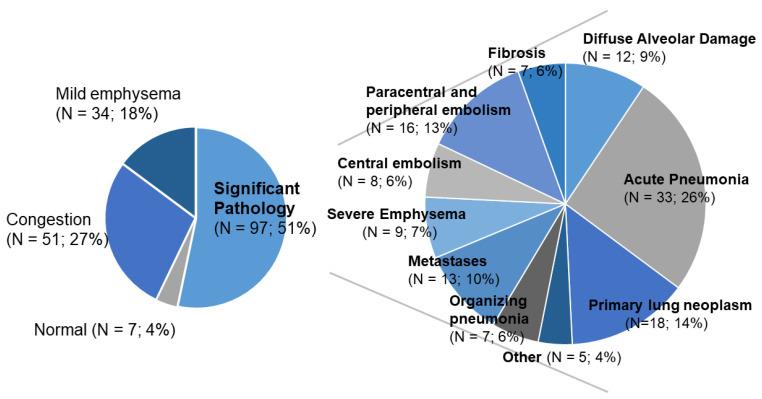
Overview of pulmonary pathologies found in consecutive adult autopsies from a 2-year period (2016–2017). A total of 197/189 patients showed significant pulmonary pathologies at postmortem examination (left side). A total of 28 patients had more than one pulmonary pathologic finding, leaving a total of 128 significant pulmonary diagnoses (right side).

The lungs were normal in 7/189 patients (4%). Sole congestion due to cardiac failure was diagnosed in 51 patients (27%) and mild emphysema in 34 patients (16%). Among those, 26 patients had both mild emphysema and congestion. These findings, however, were considered minor or non-significant primary pulmonary pathologies and were not included into further analyses. Thus, 97/189 patients (51%) presented with significant pulmonary pathology at postmortem work-up. A total of 28/189 (15%) patients had additional significant pulmonary pathology diagnoses (again, excluding congestion (N = 12) and mild emphysema (N = 51) as secondary pulmonary findings). In summary, in total, 128 significant pathologic diagnoses were finally found in 189 patients with the following frequencies: acute pneumonia was diagnosed 33/128 times (26%), embolism 24 times (19%), primary pulmonary neoplasms 18 times (14%), organizing pneumonia and other fibrosing conditions 14 times (11%), pulmonary metastases 13 times (10%), diffuse alveolar damage 12 times (9%), severe emphysema 9 times (7%) and other pathologies, e.g., amyloidosis 5/128 times (4%) (Figure 2).

With regard only to the predominant pulmonary pathological findings, there was acute pneumonia in 29 patients (15%), primary pulmonary neoplasms in 17 patients (9%), DAD in 12 patients (6%), metastases in 9 patients (5%), central embolism in 7 patients (4%), significant emphysema in 7 patients (4%), organizing pneumonia and other fibrosing conditions in 7 patients (4%), paracentral and peripheral emboli in 6 patients (3%) and other pathologies in 3 patients (2%).

### 3.3. Correlation with Demographic Data and Clinical Findings

There was no significant correlation between the presence or type of pulmonary pathology and patients’ gender (*p* = 0.87) or age (*p* = 0.44). DAD was the only predominant pulmonary diagnosis that was rendered more frequently in patients from the intensive care unit of the university hospital (7/12; 58%; *p* = 0.023). The highest lung weights were observed in DAD with a median weight of 1570 g (95% CI 1340 g–2090 g), in contrast to normal lungs with a median weight of 792 g (95% CI 560 g–1330 g).

Acute pneumonias (N = 20), pulmonary embolism (N = 10) and DAD and severe emphysema leading to cardiopulmonary failure (N = 7 each) were the most frequent causes of death associated with pulmonary pathologies and were observed in 60/189 deaths (32%). Acute pneumonias, however, were also diagnosed as additional findings in patients who died due to cerebral or cerebrovascular conditions (N = 2/7) or due to hepatic failure (N = 3/3). In contrast, in patients who died due to aortic ruptures or myocardial infarction, significant pulmonary pathologies—excluding congestion and mild emphysema—were only rarely seen (N = 1/12 for aortic rupture and N = 5/20 for myocardial infarction with 3 co-existing lung neoplasms), which was an expected finding.

### 3.4. Comparison between Clinical Diagnoses and Postmortem Findings

Comparisons of postmortem pulmonary findings with clinical diagnoses and questions revealed complete agreement in 75/189 cases (39.7%), clinical suspicion for a specific finding confirmed by postmortem examination in 57/189 cases (29.6%), unclear clinical finding and a specific finding at postmortem examination in 16/189 cases (9%), a suspicion for a specific finding not confirmed at postmortem examination in 9/189 cases (5.3%) and a specific finding at autopsy in the absence of clinical suspicion in 32 cases (16.9%). In summary, major discrepant findings regarding pulmonary pathology (i.e., the latter two categories) were found in 41/189 cases (21.7%). There was no difference between discrepancies and concordant or confirming diagnoses regarding patients’ age, gender, hospital or department (*p* > 0.2 each). The most common diagnosis with discrepancies was acute pneumonia (N = 10/29 cases discrepant), followed by metastases (N = 5/9) and paracentral/peripheral emboli (N = 4/6) when analyzing the predominant pulmonary pathologies. In contrast, primary pulmonary malignancies were almost always known (N = 16/17), as well as severe emphysema (N = 6/7) and central pulmonary emboli (N = 5/7) and organizing pneumonia or fibrosis (N = 6/7). Overall, this correlation was significant (*p* = 0.012) (Table 2). For the additional pulmonary pathologies, no association with the categories of discrepancies was found (*p* = 0.44).

## 4. Discussion

We present a comprehensive single center study on postmortem pulmonary pathology findings in 189 adults obtained during a two-year period from 2016–2017. We used a standardized method for gross preparation and histological work up of the lungs consisting in instillation of formaldehyde through the large bronchi and preparing large frontal tissue sections after proper fixation [15].

Half of the patients had one or more significant pulmonary pathology finding at postmortem examination, with pulmonary disease as cause of death in one third of the cases. Leaving out mild emphysema and congestion due to cardiac failure, the most frequent diagnoses were acute pneumonia, embolism, tumors and diffuse alveolar damage (DAD) followed by fibrosing conditions and severe emphysema. In most cases, there was a correlation between clinical and pathological diagnoses, including the confirmation of clinically suspected findings. In a low number of cases an unclear clinical finding was clarified. However, in a considerable number of patients, there were true discrepant findings regarding the lungs.

In the literature before the COVID-19 era, there are only few studies that comprehensively report postmortem pulmonary pathology data. In a smaller study from India, including 86 cases, the prevalence of lung pathology findings in autopsies was 65%. This is higher compared to our study, but the authors included congestion and edema caused by cardiac failure in their list of diagnoses [20]. Apart from that, the range of pathologies comprised mainly inflammatory diseases. In contrast to our study, surprisingly, there were no neoplasms reported. This may be explained by the very different cultural and environmental background or a referral bias. In a longitudinal epidemiologic study from Norway, the prevalence of pulmonary diseases was examined in the general population including data from autopsies, with neoplasms and pneumonia being reported as the most frequent pulmonary diseases contributing to the death of the patients [5].

In contrast, many original publications concentrate on one particular pulmonary disease complex: in one series, for example, pulmonary embolism as cause of death was studied in detail [21]. In our study, pulmonary embolism was observed in around one quarter of cases, in half of these cases contributing significantly to the death of the patients. Other studies describe histomorphological findings of patients with idiopathic pulmonary fibrosis (IPF) and observe that in the majority of patients exacerbation rather than progression leads to the fatal outcome of the disease [22,23]. In our study, the specific clinical question for IPF was asked in one case only, but there were additional cases with other fibrotic lesions, such as organizing pneumonia or severe emphysema with associated fibrosis.

Acute pneumonias, including four cases with fungal infections, were the most frequent significant pulmonary pathologies observed in this study. They were a frequent cause of death but also occurred as additional findings, e.g., in patients who died due to terminal liver failure. In these cases, however, pneumonias could well represent the underlying reason for the development of final hepatic failure in patients with liver cirrhosis. Pneumonias contributed also to the cases with discrepancies between clinical diagnosis and autopsy finding (both newly detected and non-confirmed). Comparable results on inflammatory diseases have been observed in a study on patients in intensive care units, however, without focusing on lung findings [13].

DAD was more frequently diagnosed compared to other case series, e.g., in a forensic setting [24]. DAD can not only occur as a primary disease manifestation but also as a side effect of ventilation [25] or extracorporeal membranous oxygenation, which explains the expected high rate in patients from the intensive care unit [26]. Moreover, it can occur in association with infections, most frequently viral (including, but not limited to COVID-19 [1,3,27,28]) and caused by fungi, e.g., pneumocystis jiroveci. In our case collection, DAD was observed in association with lung fibrosis, tumors, including postoperative complications and most frequently with acute pneumonia.

Studies focusing on malignancies report only few incidentally detected lung cancers [27]. Concordantly, primary lung neoplasms were not a frequent reason for diagnostic error in our study. If this occurred, it was due to rare malignancies with a challenging diagnostic course such as intravascular large B-cell lymphoma [28]. Interestingly, the primary cause of death in lung cancer patients was primarily attributable to general tumor burden, followed by secondary infections and complications of metastases [29]. In our series, we also observed cardiovascular diseases and other pulmonary pathologies as a cause of death in patients with lung malignancies.

An example of a rare pulmonary pathology finding is pulmonary amyloidosis, which was detected in 76 patients during a period of 20 years in a study from the USA [30]. In our series, we identified four cases with pulmonary amyloidosis.

In view of the current global situation, it should be emphasized that many of the pathologies observed in this study may contribute to a severe course of a COVID-19 infection, including also mild emphysema that we would have interpreted as “non-significant” pathology during the prospective collection of the cases of our study.

Many publications on autopsies focus on discrepancies between clinical and pathological diagnoses, most of them applying the Goldman Criteria [31] for the categorization of diagnostic errors. In studies of patients from intensive care units up to 20% major errors have been reported [32]. Similar rates for major diagnostic errors were also observed in a very recent, large meta-analysis [12]. We did not apply the original Goldman criteria, since they refer to the synopsis of all postmortem findings in correlation to clinical diagnoses, but used a modified system to categorize the correlations and discrepancies between pre- and postmortem findings.

Discrepancies and diagnostic errors are still rather prevalent although they are declining due to better diagnostic tools [11,12,33,34]. Pulmonary diseases, particularly pneumonia and pulmonary embolism, are amongst the most prevalent causes of diagnostic discrepancies between clinical and autopsy diagnoses—less frequent than cardiovascular events but more frequent than malignancies [6,7,8,9,12,13,18,35,36]. Consistent with that, we also observed that in 5% of the cases pulmonary pathologies contributed to true discrepancies. The value of postmortem examinations, however, is not limited to detecting the cause of death or demonstrating diagnostic errors, but encompasses clarification of unclear clinical findings and confirmation of suspected or clinically clear diagnoses. In fact, many pathological findings that we detected did not directly contribute to the fatal outcome nor were they the source of discrepancies between clinical and autopsy diagnosis, including some cases with pneumonia, emphysema, malignancies, and fibrosing lesions.

From a technical point, we think that the technique we applied allows precise macroscopic and histopathological evaluation of the lungs, with a good preservation of histology due to proper fixation and only minor artifacts caused by mechanical compression due to the good expansion of the organs. This may be of particular importance for the postmortem assessment of fibrosing diseases and of DAD. Similar to fibrosing lesions and with an overlap in organizing DAD, the evaluation of instilled and expanded airway spaces may increase the diagnostic accuracy for delicate findings such as minor hyaline membranes or may allow exact localization of fibrotic changes in the different anatomic compartments of the lung parenchyma. Since both macroscopy and histology can be performed without facing major artefacts, thus enhancing diagnostic certainty of pathological diagnoses, this approach is now considered method of choice for postmortem lung diagnostics in the institute.

There are several limitations of this study, including the presented technique. Clinical information was obtained mostly from the autopsy request forms. At this time, complete patient’s records were not routinely available before the conduction of the autopsies. While the macroscopic findings of many cases were discussed with the clinicians, only a minority of the cases were discussed in clinico-pathological conferences or multidisciplinary boards where radiologists attended the discussion. Non-availability of data, however, may contribute to diagnostic discrepancies on a systematic level [33]. Electronic patient records that are available to the pathologist may therefore also contribute to a further decline of discrepancy rates besides improved intravital diagnostics [10]. Next, the method of the preparation is labor intensive and may require a complex organization of the autopsy service. For the investigation of the complete lungs, it is necessary to keep the entire organs, which may potentially interfere with legal regulations. In Switzerland, larger tissue samples may be kept for further processing, if deemed necessary for adequate diagnosis, at the discretion of the attending staff pathologist. This may not be possible in certain countries, such as, e.g., the United Kingdom. In other countries, preserving complete organs for careful histological analysis later on may also be within a legal grey zone. Formalin instillation has to be performed exclusively through the bronchi since embolic material may be flushed further proximally by the instillation of liquid. Undirected sampling for microbiology can be performed from the outside before formalin installation, but targeted sampling is not possible. In particular cases, modification of this method or using alternative preparation approaches may be indicated, e.g., in cases where complete dissection along the pulmonary arteries and bronchi would be more useful. In view of our results and personal experience, however, we suggest that the presented preparation technique should be considered as first choice, in general or at least in the context of particular clinical questions regarding the lungs such as fibrosing diseases or acute respiratory distress syndrome corresponding to the pathologic diagnosis of DAD.

## 5. Conclusions

Taken together, this descriptive explorative study highlights the high frequency and variety of pulmonary pathologic findings obtained from postmortem investigations applying a standardized preparation technique for postmortem diagnostics of the lungs that allows excellent macroscopical and histopathological evaluation. Moreover, we could demonstrate that acute pneumonia is still a considerable cause for discrepancy between clinical and postmortem diagnostics. Correlation analysis, however, should not only focus on discrepancies, which are consistently reported in a certain number of cases. The comparison between clinical and autopsy diagnoses should also value confirmatory results in clinically ambiguous cases. A positive feedback loop in case of consistent clinical and autopsy diagnoses should not be underestimated in view of continuous medical education [33,34,37,38,39]. The recent example of COVID-19 has shown both the impact of postmortem diagnosis for the understanding of this novel disease [40,41,42,43] but has also highlighted the need of profound knowledge about general pathologic conditions, in particular, of the lungs in order to accurately interpret the findings and to separate newly discovered characteristics from well-known unspecific features. In this work, we have presented postmortem pulmonary pathology as an example of a particular organ specific aspect of autopsy or postmortem diagnostics, respectively, and we could demonstrate that this sub-discipline of pathology still fulfills the duty of quality assurance and education of medical professionals including clinicians, radiologists and pathologists.

## Figures and Tables

**Table 1:** Cause of death in consecutive adult autopsies from a 2-year period (2016–2017).

Cause of Death	N	%
Cardiopulmonal failure	54	29
(predominant respiratory component)	(18)	(10)
Myocardial infarction	21	11
Neoplasm	27	14
Pneumonia	20	11
Sepsis or peritonitis	12	6
Gastrointestinal hemorrhage	11	6
Aortic rupture	11	6
Pulmonary embolism	10	5
Cerebral/cerebrovascular	7	4
Hepatic failure	6	3
Intestinal ischemia	2	1
Others	8	4
Total	189	100

**Table 2:** Discrepant pulmonary diagnoses at postmortem investigation (main diagnoses only).

Diagnosis	Congruent (Complete Agreement/Confirmation of A Suspected Clinical Finding)	Specific Postmortem Finding of An Unclear Clinical Finding	Discrepant (No Confirmation of A Suspected Specific Finding/New Specific Finding at Autopsy)
acute pneumonia	16	3	10
metastases	4	0	5
paracentral and peripheral embolism	2	0	4
primary pulmonary neoplasm	15	1	1
congestion	42	3	6
normal	7	0	0
organizing pneumonia	2	0	1
DAD	9	1	2
severe emphysema	3	3	1
central embolism	5	0	2
mild emphysema	21	5	8
fibrosis	4	0	0
other	2	0	1
total	132	16	41
